# Supplementary material for: Mutations in PCYT2 disrupt etherlipid biosynthesis and cause a complex hereditary spastic paraplegia
Source: Brain. 2019 Oct 22;142(11):3382–97. doi: 10.1093/brain/awz291 (PMC6821184; doi:10.1093/brain/awz291)
Supplement: awz291_Supplementary_Data [file awz291_supplementary_data.zip › awz291-Suppl_data/Supplementary_Data4.zip!/Supplementary information 3 - fibroblast lipidomics.zip!/Fibroblast lipidomics/Patient1 vs Patient2/Statistical plots and data/OPLS-DA_scores.pdf]

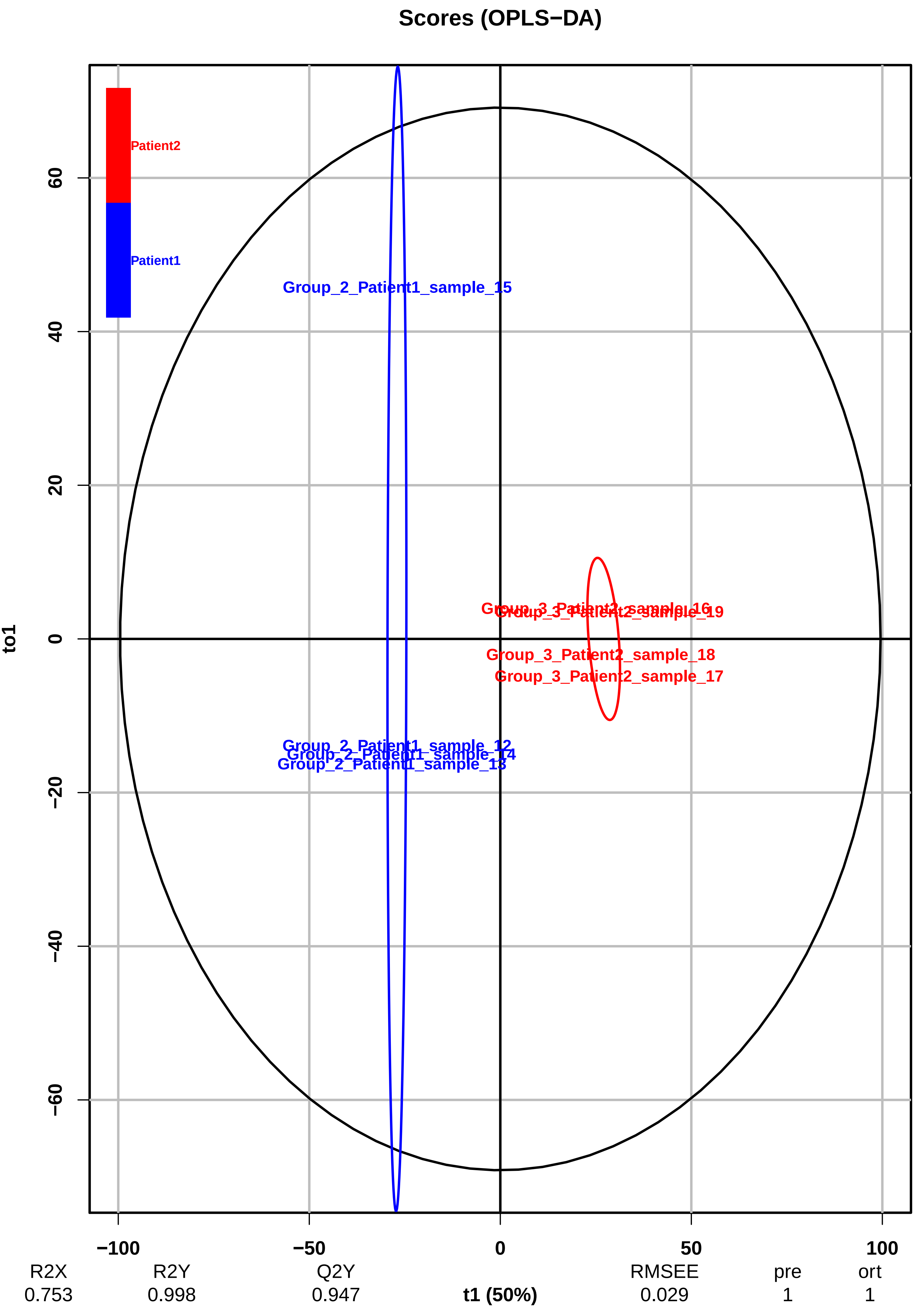
Scores (OPLS−DA)
Patient2
Patient1
Group_2_Patient1_sample_15
Group_3_Patient2_sample_16
Group_3_Patient2_sample_19
Group_3_Patient2_sample_18
Group_3_Patient2_sample_17
Group_2_Patient1_sample_12
Group_2_Patient1_sample_14
Group_2_Patient1_sample_13
60
40
20
0
−20
−40
−60
to1
−100
−50
0
50
100
t1 (50%)
R2X 0.753
R2Y 0.998
Q2Y 0.947
RMSEE 0.029
pre 1
ort 1
